# The Traditional Kampo Medicine Tokishakuyakusan Increases Ocular Blood Flow in Healthy Subjects

**DOI:** 10.1155/2014/586857

**Published:** 2014-04-24

**Authors:** Shin Takayama, Yukihiro Shiga, Taiki Kokubun, Hideyuki Konno, Noriko Himori, Morin Ryu, Takehiro Numata, Soichiro Kaneko, Hitoshi Kuroda, Junichi Tanaka, Seiki Kanemura, Tadashi Ishii, Nobuo Yaegashi, Toru Nakazawa

**Affiliations:** ^1^Comprehensive Education Center for Community Medicine, Tohoku University Graduate School of Medicine, 2-1 Seiryo-Machi, Aoba Ward, Sendai City 980-8575, Japan; ^2^Department of Education and Support for Community Medicine, Tohoku University Hospital, 1-1 Seiryo-Machi, Aoba Ward, Sendai City 980-8574, Japan; ^3^Department of Ophthalmology, Tohoku University Graduate School of Medicine, 1-1 Seiryo-Machi, Aoba Ward, Sendai City 980-8574, Japan; ^4^Department of Obstetrics and Gynecology, Tohoku University Graduate School of Medicine, 1-1 Seiryo-Machi, Aoba Ward, Sendai City 980-8574, Japan; ^5^Division of General Medicine, Saitama Medical Center, Jichi Medical University, 1-847 Amanumacho, Omiya Ward, Saitama City 330-8503, Japan

## Abstract

The aim of this study was to examine the effects of oral administration of kampo medical formulas on ocular blood flow (OBF). A crossover protocol was used to randomly administer five grams of yokukansan, tokishakuyakusan (TSS), keishibukuryogan, or hachimijiogan to 13 healthy blinded subjects (mean age: 37.3 ± 12.3 years). The mean blur rate, a quantitative OBF index obtained with laser speckle flowgraphy, was measured at the optic nerve head before and 30 minutes after administration. Blood pressure (BP) and intraocular pressure (IOP) were also recorded. No significant changes were observed in mean BP or IOP after the administration of any of the kampo medical formulas. There was a significant increase in OBF 30 minutes after administration of TSS (100% to 103.6 ± 6.9%, *P* < 0.01). Next, TSS was administered to 19 healthy subjects (mean age: 32.0 ± 11.0 years) and OBF was measured before and 15, 30, 45, and 60 minutes after administration. Plain water was used as a control. OBF increased significantly after TSS administration compared to control (*P* < 0.01) and also increased from 30 to 60 minutes after administration compared to baseline (*P* < 0.05). These results suggest that TSS can increase OBF without affecting BP or IOP in healthy subjects.

## 1. Introduction

Ocular blood flow (OBF) abnormalities have been reported to play an important role in the pathogenesis of many ocular diseases, including glaucoma and diabetic retinopathy [1–4]. An understanding of these abnormalities is therefore of critical importance in determining the pathophysiological features of these diseases and finding novel treatments.

Japanese kampo medicine has a history spanning more than 1500 years. Kampo medical formulas have been used to treat a variety of physical illnesses and diseases [5], including ocular conditions such as dry eye, blurred vision, decreased visual acuity, and visual field defects [6]. However, the clinical efficacy of kampo medicine has traditionally been evaluated with subjective assessments of symptoms, and there is little quantitative information on the effects of kampo medical formulas on the eye [7]. A few such studies have suggested that yokukansan (YKS) has an influence on blood flow in the short posterior ciliary artery in rabbits [8] and that hachimijiogan (HJG) has an effect on blood flow in the central retinal artery in humans [9]. Other studies have reported that tokishakuyakusan (TSS) has an inhibitory effect on platelet aggregation and a relaxative effect on vascular smooth muscle [10, 11]. Additionally, a study of erythrocyte aggregability and deformability found that keishibukuryogan (KBG) had an influence on microcirculation [12, 13]. These four kampo medical formulas thus have the potential to influence ocular circulation, but so far, no studies have been conducted to evaluate their effects.

Recently, we have reported that laser speckle flowgraphy (LSFG) and color Doppler imaging can be used to assess the effects of topical medications or acupuncture on OBF, including circulation in the optic nerve head (ONH) and retrobulbar space [14–16]. LSFG allows us to quantify microcirculation in the ONH, the choroid, and the retina simultaneously [17]. It is a noncontact technique based on the laser speckle phenomenon and has been shown to be reliable and reproducible in human eyes, especially in the ONH [18–20]. Furthermore, it can acquire an image of OBF in just a few seconds [17]. We believe that these characteristics make LSFG a suitable instrument for this study's evaluation of dynamic OBF alterations in healthy eyes after the administration of kampo formulas.

Thus, the object of this study was to investigate, with healthy volunteers and LSFG, the effects on OBF of kampo formulas (YKS, TSS, KBG, and HJG) traditionally used for the treatment of eye disease.

## 2. Methods

### 2.1. Experiment  1 (Ex.  1)

#### 2.1.1. Subjects

Healthy, nonsmoking volunteers ranging from 20 to 70 years old were recruited from respondents to a poster campaign at Tohoku University Hospital, Miyagi, Japan, between April 2013 and November 2013. Subjects were included in this experiment if baseline intraocular pressure (IOP) was below 22 mmHg in both eyes in a Goldmann applanation tonometry examination, findings were normal in slit lamp and funduscopic examinations, and refractive error was within a range of −8.5 to −0.5 diopters (mean of the included subjects: −2.2 ± 2.0). Subjects were excluded if they had a history of ophthalmic or general disorders, had ocular laser or incisional surgery in either eye, or were receiving systemic or topical medication. Subjects abstained from alcohol and caffeine for at least six hours before the measurements. Both eyes of each participant were monitored. The procedures in all experiments followed the tenets of the Declaration of Helsinki and were approved by the committee of Tohoku University Graduate School of Medicine. Written informed consent was obtained from all participants. Overall, 26 eyes of 13 healthy volunteers (mean age, 37.3 ± 12.3 years; 6 men and 7 women) were included in this experiment.

#### 2.1.2. Study Design

This was a randomized, double-blinded, crossover experiment.

#### 2.1.3. Randomization

No participants or examiners had any knowledge of kampo medicine and they were blinded to the order of administration of the formulas, which was randomized. The washout period between administrations of the formulas was at least one week. All four formulas were administered to each participant over the course of two months.

#### 2.1.4. Measurement of Clinical Parameters

IOP was determined with Goldmann applanation tonometry. Both systemic blood pressure (BP) and pulse rate (PR) were conventionally measured from the left brachial artery at the height of the heart with an automated BP monitor (HEM-759 E, Omron Corporation, Kyoto, Japan). Mean arterial blood pressure (MBP) was calculated from systolic BP (SBP) and diastolic BP (DBP) according to the following formula: MBP = DBP + 0.42 (SBP−DBP) [21–23].

#### 2.1.5. LSFG

The principles of LSFG have previously been described in detail [24, 25]. Briefly, this instrument consists of a fundus camera equipped with a diode laser (830 nm wavelength) and an ordinary charge-coupled device sensor (750 × 360 pixels). This study used the LSFG-NAVI device (Softcare Co., Ltd., Fukutsu, Japan), which has been approved by the Pharmaceuticals and Medical Devices Agency in Japan. Mean blur rate (MBR), a measurement of relative OBF expressed in arbitrary units, is determined using the pattern of speckle contrast produced by the interference of a laser scattered by blood cells moving in the ocular fundus [26]. The MBR images are acquired continuously at the rate of 30 frames per second over a 4-second period. The accompanying analysis software (LSFG Analyzer, version 3.0.43.0) combines all captured images into a composite map of OBF. In this study, we identified the ONH margin and set this area as the region of interest in the software. The software then automatically calculated the MBR in the ONH from the OBF map, as we have described in a previous report [16].

#### 2.1.6. Intervention

One of the four kampo medical formulas was prepared in 50 mL of plain hot water and orally administered to the subject. The kampo medical formulas included YKS, TSS, KBG, and HJG. All were produced by Tsumura and Co. (Tokyo, Japan). The compositions of the formulas, which are all mixtures of dried herbs registered in the Pharmacopoeia of Japan, are as follows. YKS contains 4.0 g Atractylodis Lanceae Rhizoma, 4.0 g Poria, 3.0 g Cnidii Rhizoma, 3.0 g Angelicae Radix, 2.0 g Bupleuri Radix, 1.5 g Glycyrrhizae Radix, and 3.0 g Uncariae Uncis Cum Ramulus; TSS contains 4.0 g Paeoniae Radix, 4.0 g Atractylodis Lanceae Rhizoma, 4.0 g Alismatis Rhizoma, 4.0 g Poria, 3.0 g Cnidii Rhizome, and 3.0 g Angelicae Radix; KBG contains 3.0 g Cinnamomi Cortex, 3.0 g Paeoniae Radix, 3.0 g Persicae Semen, 3.0 g Poria, and 3.0 g Moutan bark; and HJG contains 6.0 g Rehmanniae Radix, 3.0 g Corni Fructus, 3.0 g Dioscoreae Rhizoma, 3.0 g Alismatis Rhizoma, 3.0 g Poria, 2.5 g Moutan Cortex, 1.0 g Cinnamomi Cortex, and 0.5 g Aconiti Tuber.

#### 2.1.7. Testing Protocol

All tests were performed between 6:00 and 8:00 p.m. at an ambient room temperature of 25 degrees Celsius. On the day of the test, after a slit lamp examination, 0.4% tropicamide (Mydrin M; Santen Pharmaceutical Co., Ltd., Osaka, Japan) was used to dilate the pupil. All subjects received a funduscopic examination, and IOP was measured with Goldmann applanation tonometry. To ensure that systemic hemodynamic conditions in each subject were consistent, all subjects rested in a sitting position for 10 minutes before the start of the investigation, in accordance with previous studies [16–20]. Immediately after the 10-minute rest period, systemic BP, PR, and IOP were recorded, and triplicate measurements of OBF were made with LSFG. The formula was administered, and after 30 minutes, triplicate OBF measurements were made again. The entire procedure took place in a darkened room. Normalized MBR was used for the statistical analysis.

#### 2.1.8. Statistical Analysis

The analysis used percentage values (%) calculated by defining the baseline measurement variables as 100%. The values were the mean ± standard deviation. Measurements of baseline variables made on different days were compared with the Kruskal-Wallis test. The Wilcoxon signed-rank test was used to assess differences in the values before and after kampo administration. All statistical analyses were performed with JMP software (Pro version 10.0.2; SAS Institute Japan, Inc., Tokyo, Japan). The difference was considered significant when the *P* value was less than 0.05.

### 2.2. Experiment  2 (Ex.  2)

#### 2.2.1. Subjects

The inclusion criteria, recruitment of subjects, and procedures of the test were the same as those in Ex.  1. Overall, 38 eyes of 19 healthy volunteers (mean age: 32.0 ± 11.0 years, male : female = 8 : 11) were included in this experiment.

#### 2.2.2. Study Design

This was a double-blinded, controlled experiment.

#### 2.2.3. Measurement of Clinical Parameters

Measurement of IOP, BP, PR, and OBF was the same as in Ex.  1.

#### 2.2.4. Intervention

Five grams of TSS prepared in 50 mL of hot water was administered to each subject. As a control, 50 mL of plain hot water was also administered to each subject on a different day, with a washout period of at least one week between the administration of TSS and water.

#### 2.2.5. Kampo Diagnostic Questionnaire

A questionnaire was given to the subjects to determine their scores for “qi,” “blood,” and “fluid,” traditional diagnostic criteria used in kampo medicine. The questionnaire was obtained from a kampo manual written by Terasawa [27]. “Qi,” “blood,” and “fluid” are considered fundamental components of the human body in kampo medicine, and disorders and disease are associated with imbalances of these components. These imbalances are classified as “qi deficiency,” “qi stagnation,” “qi counter flow,” “blood deficiency,” “blood stasis,” and “fluid retention.” [28].

#### 2.2.6. Testing Protocol

The procedures of the test were the same as in Ex.  1. Systemic BP, PR, and OBF were measured just before and 15, 30, 45, and 60 minutes after administration of TSS or water. IOP was recorded before and 60 minutes after administration.

#### 2.2.7. Statistical Analysis

The determination of baseline values was with the same method as in Ex.  1. LSFG values after administration of TSS and water were compared at each time point with a two-way analysis of variance (ANOVA). The repeated ANOVA values and post hoc Dunnett's test were used for a statistical comparison of baseline and postadministration values. Differences were considered significant with a *P* value of less than 0.05.

## 3. Results

### 3.1. Ex.  1

#### 3.1.1. Comparison of Baseline Variables of the Subjects

Table 1 summarizes the measurements of the baseline variables in the subjects before the administration of each formula. There were no significant differences in OBF, IOP, SBP, DBP, MBP, or PR between the subjects before they were administered any of the formulas.

#### 3.1.2. Alteration of Systemic Variables, IOP, and OBF after Administration of Each Kampo Formula

No significant changes were observed in IOP or MBP after the administration of any of the kampo formulas. PR significantly decreased after the administration of YKS, TSS, and HJG (Table 2). Thirty minutes after the administration of TSS, LSFG measurements of OBF revealed significant increases (100% to 103.6 ± 6.9%, *P* < 0.01). By contrast, 30 minutes after the administration of YKS, KBG, or HJG there were no significant changes in OBF. Additionally, there were no adverse events from administration of any of the kampo medical formulas during the experiment.

### 3.2. Ex.  2

#### 3.2.1. Comparison of Baseline Variables in the Subjects

Table 3 summarizes the baseline variables of the subjects before administration of water or TSS. There were no significant differences in OBF, SBP, DBP, MBP, or PR, before the administration of either substance, although there was a small, though statistically significant, difference in IOP.

#### 3.2.2. Alteration in OBF after Administration

**Figure 1:**
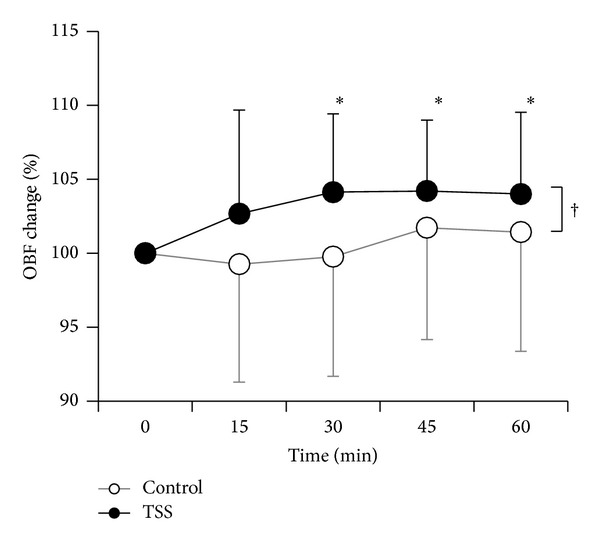
Dynamic OBF changes in response to administration of TSS (●) and control (○). Data are expressed as mean ± standard deviation. The dagger indicates a statistically significant difference between TSS and control (two-way analysis of variance; ANOVA). The asterisk indicates a statistically significant difference from the baseline (repeated measurements of ANOVA, with post hoc Dunnett's test).

**Figure 2:**
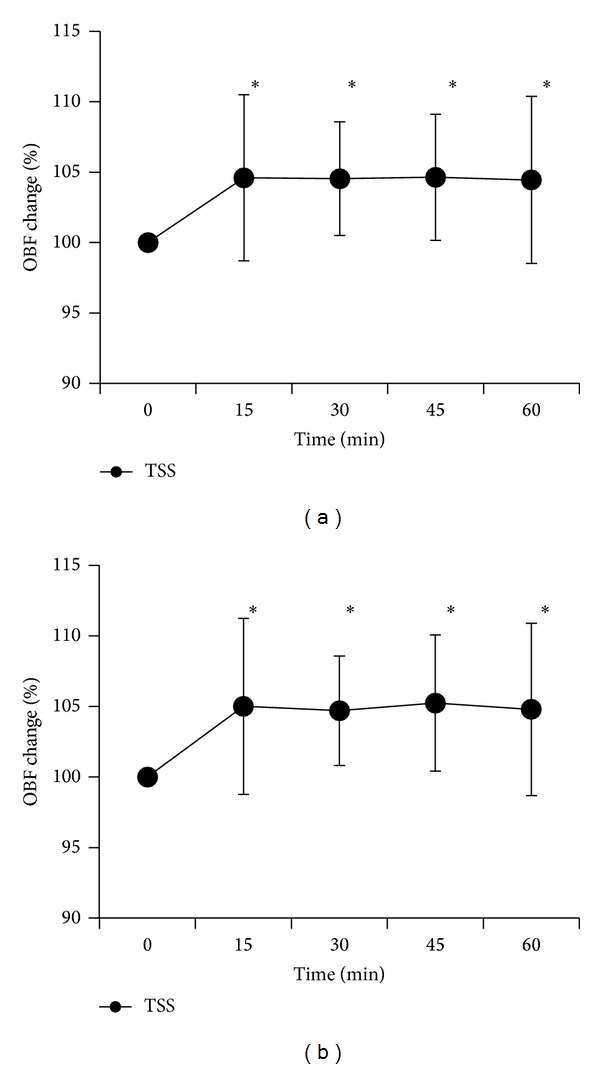
Dynamic OBF changes in subjects with blood deficiency and blood stasis (a) and with blood deficiency and fluid retention (b) in response to administration of TSS. Data are expressed as mean ± standard deviation. The asterisk indicates a statistically significant difference from the baseline (repeated measurements of ANOVA, with post hoc Dunnett's test).

**Figure 3:**
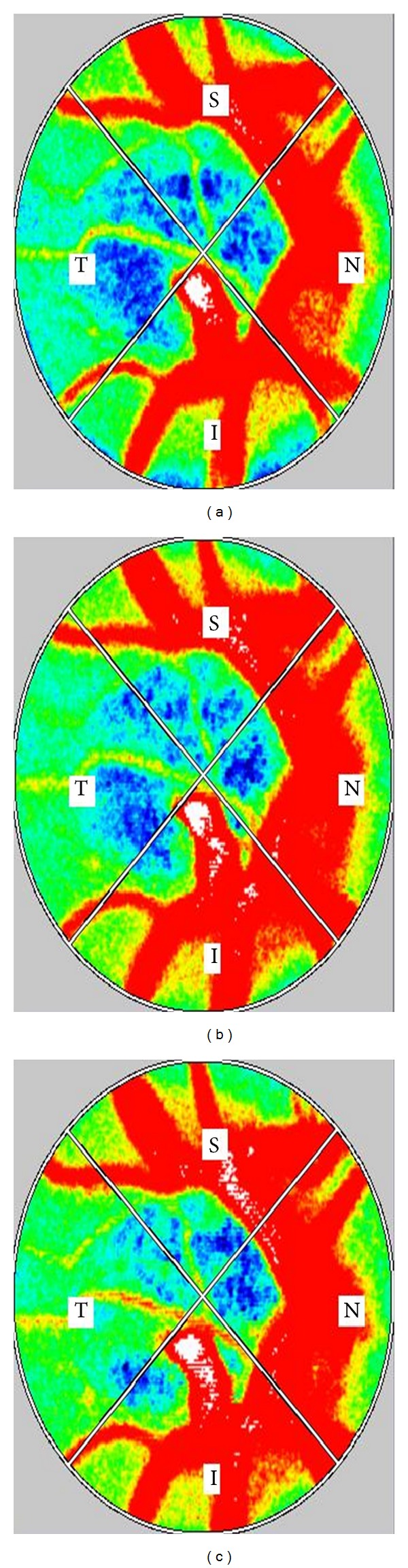
Representative MBR images of the entire ONH before administration and 30 and 60 minutes after the administration of TSS in healthy subjects (right eye). (a) Composite blood flow map of the ONH before the administration of TSS. The MBR value is 21.2. (b) Composite blood flow map of the ONH 30 minutes after the administration of TSS. The MBR value is 24.8. (c) Composite blood flow map of the ONH 60 minutes after the administration of TSS. The MBR value is 26.1.

OBF after the administration of TSS or water was significantly different (*P* < 0.01) (Table 4). OBF increased significantly 30, 45, and 60 minutes after administration of TSS (100% to 104.1 ± 5.3%; *P* < 0.05, 104.2 ± 4.8%; *P* < 0.05, 104.0 ± 5.5%; *P* < 0.05) (Figure 1). According to the results of the kampo diagnostic questionnaire, 5 subjects had “qi deficiency,” 3 subjects had “qi stagnation,” 6 subjects had “qi counter flow,” 4 subjects had “blood deficiency,” 10 subjects had “blood stasis,” and 8 subjects had “fluid retention.” OBF increased significantly 15 to 60 minutes after the administration of TSS in the group of subjects with “blood deficiency” and “blood stasis” (15 minutes, *P* < 0.05; 30 minutes, *P* < 0.05; 45 minutes, *P* < 0.05; 60 minutes, *P* < 0.05) and in the group with “blood deficiency” and “fluid retention” (15 minutes, *P* < 0.05; 30 minutes, *P* < 0.05; 45 minutes, *P* < 0.05; 60 minutes, *P* < 0.05) (Figures 2(a) and 2(b)).  Figure 3 shows a representative image of OBF in the ONH before administration of TSS, as well as 30 and 60 minutes after administration.

## 4. Discussion

Ex.  1 revealed that, of four candidate kampo formulas, only TSS had the effect of increasing OBF. This was somewhat in contrast to previous studies, which have found that YKS has an influence on blood flow in the short posterior ciliary artery in rabbits [8] and that HJG has an effect on blood flow in the central retinal artery in humans [9]. This may be due to differences in OBF measurement technique and location between these previous studies and the present one. The previous studies evaluated OBF from the retrobulbar side, whereas this study evaluated OBF from the ocular side, in the ONH.

The mechanism of the effects of TSS and KBG on circulation has been described in several reports. Nasu et al. reported that TSS had an inhibitory effect on platelet aggregation [10]. KBG has been reported to improve endothelial function in patients with metabolic syndrome [29]. Additionally, an evaluation of erythrocyte aggregability and deformability in patients with multiple old lacunar infarctions found that KBG improved microcirculation [12, 13]. TSS has a number of components in common with YKS, KBG, and HJG. Atractylodis Lanceae Rhizoma, included in YKS and TSS, has been reported to inhibit platelet aggregation [10]. Poria, included in YKS, TSS, KBG, and HJG, has been reported to have a similar effect [10]. Paeoniae Radix, included in TSS and KBG, has been reported to relax vascular smooth muscle [11]. Polyphenols of Cinnamomi Cortex or cinnamaldehyde, included in KBG and HJG, have been reported to have an endothelium-dependent relaxative effect [30–33]. Moreover, these four kampo formulas include multiple physiologically active substances that have yet to be fully identified. The interactions between them are also not yet fully understood.

In Ex.  2, we found that TSS increased OBF without affecting BP or IOP. This additional experiment supported the preliminary results of Ex.  1. In kampo practice, TSS is used to treat “blood deficiency,” “fluid retention,” and “blood stasis” [34], which are part of six conditions used in kampo practice and described by Terasawa. These conditions also include “qi deficiency,” “qi stagnation,” and “qi counter flow.” Classification of patients depends on their symptoms and a physical examination [27, 28, 34]. “Blood deficiency” and “blood stasis” are believed to be associated with general symptoms of eye disease such as blurred vision, decreased visual acuity, and visual field defects, while increased IOP is believed to be associated with “fluid retention” and “qi stagnation.” Kampo formulas are chosen based on the patient's symptoms and the results of a physical examination in a clinical setting, a methodology that has been in use since ancient times. Patients with combined “blood deficiency” and “blood stasis” or combined “blood deficiency” and “fluid retention” are considered suitable cases for treatment with TSS in kampo practice, and in Ex.  2 of this study, the OBF increase was in fact most remarkable in subjects with these conditions. This result supports the existence of a relationship between the effectiveness of kampo formulas in practice and diagnoses made according to kampo theory. The effectiveness of the formulas has traditionally been judged only by subjectively measured improvements, with quantitative and objective reports of the effects of kampo formulas on ocular circulation being very limited. The present study sought to alleviate this deficiency. We believe our results indicate that, to be most effective, kampo formulas should be chosen after patients are evaluated by both traditional kampo diagnostic practices and western medical techniques.

The authors have previously reported that 60 minutes after administration of topical tafluprost, LSFG measurements of OBF increased significantly in the eyes of normal subjects and patients with normal tension glaucoma (NTG) (100% to 104.3 ± 6.6%, *P* < 0.01) [16]. This study revealed that TSS had a similar ability to significantly increase OBF in a similar timeframe. In addition, while tafluprost led to a significant decrease in IOP, TSS had no effect on IOP. Recently, it has been suggested that impaired ocular circulation may contribute to the progression of glaucomatous damage [35, 36]. New drugs or interventions to improve ocular hemodynamics have thus become a promising target of research. Hypotension often coincides with NTG [37], and as oral vasodilators are difficult to use, to both improve OBF and treat hypotension, TSS administration, alone and in combination with topical medications such as tafluprost, may be an effective strategy to improve fundus circulation in glaucoma patients, especially those with NTG.

*Limitations*. We found that PR decreased after the administration of YKS, TSS, KBG, and HJG. Moreover, this decrease occurred in all groups of subjects including those who were administered a control substance, leading us to believe that it was a nonspecific response. The subjects waited in a quiet, darkened room for over 30 minutes, and the observed decrease in PR may have been an effect of relaxation. The other limitation of this study was the small number of healthy subjects included and the short observation time. In the future, we hope to conduct a longitudinal clinical study to determine the effectiveness of TSS in patients with eye disease, especially NTG.

## 5. Conclusions

TSS increased OBF without affecting BP or IOP in healthy subjects. Further study will be needed to investigate the effect of TSS in patients with eye disease.

## Figures and Tables

**Table 1:** Baseline variables in the four study groups (Experiment  1).

Variable	YKS	TSS	KBG	HJG	*P* value
OBF (AU)	24.5 ± 3.5	22.8 ± 2.8	23.4 ± 3.8	23.6 ± 3.8	0.48
IOP (mmHg)	12.9 ± 2.5	14.2 ± 2.4	13.6 ± 2.5	13.2 ± 2.7	0.28
SBP (mmHg)	109.7 ± 10.9	113.3 ± 10.9	111.8 ± 9.2	112.0 ± 14.4	0.95
DBP (mmHg)	64.8 ± 12.0	66.6 ± 9.9	67.3 ± 10.3	64.4 ± 7.6	0.75
MBP (mmHg)	83.7 ± 10.7	85.4 ± 10.0	86.0 ± 9.0	84.4 ± 9.8	0.90
PR (beats/min)	73.6 ± 7.7	75.5 ± 10.7	71.0 ± 8.2	77.2 ± 6.8	0.28

Data are expressed as mean ± standard deviation. Differences between groups were assessed with the Kruskal-Wallis test.

**Table 2:** Alteration of IOP and systemic variables in response to administration of each kampo formula (Experiment  1).

	Variable	Baseline	30 minutes after treatment	*P* value
YKS	OBF change (%)	100.0 ± 0.0	96.9 ± 6.7	0.05
	IOP (mmHg)	12.9 ± 2.5	13.0 ± 2.9	0.73
	MBP (mmHg)	83.7 ± 10.7	87.8 ± 8.2	0.13
	PR (beats/min)	73.6 ± 7.7	66.9 ± 7.8	<0.01

TSS	OBF change (%)	100.0 ± 0.0	103.6 ± 6.9	<0.01
	IOP (mmHg)	14.2 ± 2.4	14.1 ± 2.5	0.98
	MBP (mmHg)	85.4 ± 10.0	86.8 ± 4.9	0.27
	PR (beats/min)	75.5 ± 10.7	67.0 ± 8.0	<0.01

KBG	OBF change (%)	100.0 ± 0.0	101.8 ± 6.9	0.35
	IOP (mmHg)	13.6 ± 2.5	13.3 ± 2.9	0.17
	MBP (mmHg)	86.0 ± 9.0	86.9 ± 8.9	0.74
	PR (beats/min)	71.0 ± 8.2	66.6 ± 7.9	0.05

HJG	OBF changc (%)	100.0 ± 0.0	100.0 ± 6.9	0.89
	IOP (mmHg)	13.2 ± 2.7	13.1 ± 2.5	0.81
	MBP (mmHg)	84.4 ± 9.8	85.9 ± 6.0	0.74
	PR (beats/min)	77.2 ± 6.8	67.2 ± 7.7	<0.01

Data are expressed as mean ± standard deviation. The Wilcoxon signed-rank test was applied to assess differences in the values before and after kampo administration.

**Table 3:** Baseline variables in the 2 study groups (Experiment  2).

Variable	Control	TSS	*P* value
OBF (AU)	25.1 ± 4.3	24.7 ± 4.6	0.25
IOP (mmHg)	12.8 ± 2.2	13.8 ± 2.0	<0.01
SBP (mmHg)	106.2 ± 11.4	110.1 ± 14.2	0.16
DBP (mmHg)	64.9 ± 8.4	67.9 ± 10.8	0.09
MBP (mmHg)	82.3 ± 9.1	85.7 ± 11.7	0.11
PR (beats/min)	73.1 ± 13.1	73.2 ± 9.5	0.65

Data are expressed as mean ± standard deviation. Differences between groups were assessed with the Kruskal-Wallis test.

**Table 4:** Alteration of ocular blood flow and clinical variables in response to administration of kampo formula (Experiment  2).

	Variable	Baseline	15 minutes after treatment	30 minutes after treatment	45 minutes after treatment	60 minutes after treatment	*P* value
Control	OBF change (%)	100.0 ± 0.0	99.3 ± 8.0	99.8 ± 8.1	101.7 ± 7.6	101.4 ± 8.1	0.49
	IOP (mmHg)	12.8 ± 2.2				12.4 ± 2.1	0.11
	SBP (mmHg)	106.2 ± 11.4	106.9 ± 11.5	106.2 ± 10.2	108.0 ± 13.0	108.1 ± 13.5	0.98
	DBP (mmHg)	64.9 ± 8.4	67.5 ± 12.0	65.3 ± 7.4	65.8 ± 7.4	66.9 ± 8.4	0.90
	MBP (mmHg)	82.3 ± 9.1	84.1 ± 11.0	82.5 ± 8.3	83.5 ± 9.0	84.2 ± 10.0	0.96
	PR (beats/min)	73.1 ± 13.1	66.7 ± 8.1	65.7 ± 7.5	67.7 ± 7.8	66.3 ± 8.1	0.12

TSS	OBF change (%)	100.0 ± 0.0	102.7 ± 7.0	104.1 ± 5.3	104.2 ± 4.8	104.0 ± 5.5	<0.01
	IOP (mmHg)	13.8 ± 2.0				13.6 ± 1.8	0.41
	SBP (mmHg)	110.1 ± 14.2	110.1 ± 10.0	108.6 ± 10.3	108.3 ± 11.5	109.0 ± 10.7	0.98
	DBP (mmHg)	67.9 ± 10.8	69.3 ± 9.8	68.3 ± 8.4	69.3 ± 9.2	71.0 ± 10.8	0.90
	MBP (mmHg)	85.7 ± 11.7	86.4 ± 9.1	85.2 ± 8.5	85.7 ± 9.1	87.0 ± 10.1	0.45
	PR (beats/min)	67.9 ± 10.8	69.3 ± 9.8	68.3 ± 8.4	69.3 ± 9.2	71.0 ± 10.8	0.19

Data are expressed as mean ± standard deviation. The Wilcoxon signed-rank test was applied to assess differences in IOP before and after kampo administration. A one-way analysis of variance was used to assess differences in other values before and after kampo administration.

## Abbreviations

OBF: Ocular blood flow
YKS: Yokukansan
TSS: Tokishakuyakusan
KBG: Keishibukuryogan
HJG: Hachimijiogan
LSFG: Laser speckle flowgraphy
ONH: Optic nerve head
Ex.: Experiment
IOP: Intraocular pressure
BP: Blood pressure
PR: Pulse rate
MBP: Mean arterial blood pressure
SBP: Systolic BP
DBP: Diastolic BP
MBR: Mean blur rate
NTG: Normal tension glaucoma.
